# Toxicity of benthic dinoflagellates on grazing, behavior and survival of the brine shrimp *Artemia salina*

**DOI:** 10.1371/journal.pone.0175168

**Published:** 2017-04-07

**Authors:** Raquel A. F. Neves, Tainá Fernandes, Luciano Neves dos Santos, Silvia M. Nascimento

**Affiliations:** 1 Laboratório de Microalgas Marinhas, Departamento de Ecologia e Recursos Marinhos, Instituto de Biociências, Universidade Federal do Estado do Rio de Janeiro (UNIRIO), Rio de Janeiro, Brazil; 2 Laboratório de Ictiologia Teórica e Aplicada, Departamento de Ecologia e Recursos Marinhos, Instituto de Biociências, Universidade Federal do Estado do Rio de Janeiro (UNIRIO), Rio de Janeiro, Brazil; 3 Programa de Pós-Graduação em Biodiversidade Neotropical (PPGBIO), Universidade Federal do Estado do Rio de Janeiro (UNIRIO), Rio de Janeiro, Brazil; Stockholm University, SWEDEN

## Abstract

Harmful algae may differently affect their primary grazers, causing sub-lethal effects and/or leading to their death. The present study aim to compare the effects of three toxic benthic dinoflagellates on clearance and grazing rates, behavioral changes, and survival of *Artemia salina*. Feeding assays consisted in 1-h incubations of brine shrimps with the toxic *Prorocentrum lima*, *Gambierdiscus excentricus* and *Ostreopsis* cf. *ovata* and the non-toxic *Tetraselmis* sp. Brine shrimps fed unselectively on all toxic and non-toxic algal preys, without significant differences in clearance and ingestion rates. Acute toxicity assays were performed with dinoflagellate cells in two growth phases during 7-h to assess differences in cell toxicity to *A*. *salina*. Additionally, exposure to cell-free medium was performed to evaluate its effects on *A*. *salina* survival. The behavior of brine shrimps significantly changed during exposure to the toxic dinoflagellates, becoming immobile at the bottom by the end of the trials. Dinoflagellates significantly affected *A*. *salina* survival with 100% mortality after 7-h exposure to cells in exponential phase (all treatments) and to *P*. *lima* in stationary phase. Mortality rates of brine shrimps exposed to *O*. cf. *ovata* and *G*. *excentricus* in stationary phase were 91% and 75%, respectively. However, incubations of the brine shrimps with cell-free medium did not affect *A*. *salina* survivorship. Significant differences in toxic effects between cell growth phases were only found in the survival rates of *A*. *salina* exposed to *G*. *excentricus*. Acute exposure to benthic toxic dinoflagellates induced harmful effects on behavior and survival of *A*. *salina*. Negative effects related to the toxicity of benthic dinoflagellates are thus expected on their primary grazers making them more vulnerable to predation and vectors of toxins through the marine food webs.

## Introduction

Marine benthic dinoflagellates are important primary producers and most of their representatives are potentially toxic [1]. Recently, this group of organisms has received significant scientific attention since the occurrence of benthic harmful algal blooms (HAB) has increased worldwide [1]. Among benthic dinoflagellates, blooms of *Ostreopsis* cf. *ovata* have been recorded with increasing frequency, intensity and distribution, particularly in the Mediterranean Sea [2–3], with adverse consequences on benthic communities and human intoxication, mainly through the inhalation of marine aerosols [4]. In Brazil, blooms of *O*. cf. *ovata* became a recurrent event since 1998 in Rio de Janeiro state [5] with high mortalities of the sea urchin *Echinometra lucunter* reported during these HAB events [6]. Similar ecological effects were observed in New Zealand, where blooms of *O*. *siamensis* caused decline in the numbers of the sea urchin *Evechinus chloroticus* [7]. *Ostreopsis* cf. *ovata* produces ovatoxins, and other PLTX analogues, one of the most toxic molecules occurring in nature that cause intoxication in humans [8]. There is evidence of PLTX and its analogues presence in crustaceans, molluscs and fish that, when contaminated, can cause the clupeotoxism disease by the consumption of sardines and anchovies (clupeoid fish) [4]. Moreover, a cytotoxic non-palytoxin derivative recently isolated from *O*. cf. *ovata*, ostreol A, was shown to have *in vitro* cytotoxicity against the brine shrimp *Artemia salina* [9].

*Prorocentrum lima* is a cosmopolitan species and often constitutes a significant part of benthic dinoflagellate communities worldwide [10]. This dinoflagellate produces okadaic acid (OA) and dinophysistoxins (DTXs), the main toxins responsible for diarrheic shellfish poisoning [11]. Evidence shows that every culture of *P*. *lima* tested to date has been found to produce OA and its analogues in varying quantities [12]. In Rio de Janeiro, *P*. *lima* is found all year round growing epiphytically on macroalgae and locally isolated strains demonstrated the production of high OA concentrations [13]. There is some evidence that *P*. *lima* can act as a vector for DSP toxins in shellfish and suspected cases of intoxication have been recorded in Argentina [14], Canada [15], the United Kingdom [16] and Japan [17].

*Gambierdiscus excentricus* produces maitotoxins (MTXs) and ciguatoxins (CTX) responsible for ciguatera fish poisoning [18], a disease caused by the consumption of herbivorous and carnivorous fish that have accumulated CTX through the food web. It is estimated that 50.000 to 500.000 people are affected by CTX every year, and ciguatera is the most frequently reported non-bacterial illness associated with seafood consumption worldwide [19]. Moreover, reef disturbance by hurricanes, military and tourist developments, as well as coral bleaching and the rise in water temperatures are increasing the risk of ciguatera by freeing up space for the growth of macroalgae in which *Gambierdiscus* colonize upon [20].

Despite some grazers may avoid certain toxic algae [21–22], most benthic species feed indiscriminately on toxic cells. Effects of algal toxins on their primary grazers are variable [23]. Zooplankton species may be affected by toxins showing sub-lethal symptoms such as changes in pulsation frequency and immobility [24], grazing and motility suppression [25], and even lethal responses [26–27]. Moreover, HAB events may lead to shifts in community composition to more resistant species, generating complex cascading effects through the pelagic and benthic food webs [28]. Contaminated individuals may also transfer phycotoxins through the marine trophic web by the direct predation [29–30], by the elimination of toxic cells in biodeposits and feces and through the release of toxins after their death making toxins available for detritivorous species [31–33].

Most information on interactions between toxic algae and their grazers comprises phytoplankton and zooplankton grazers, particularly copepods [23, 34–35]. Differential survival rates of toxin-exposed individuals may indicate some degree of toxin resistance by individuals selected in natural populations in the long-term [36, 37]. From an ecological point of view, toxin-resistant zooplankton fed harmful alga are more hazardous than sensitive individuals by its higher capacity for toxin accumulation which favors toxin transfer to higher consumers and dispersion of toxins through marine environments. Thus, the interactions between benthic harmful algae and their grazers must be better investigated in order to assess the potential ecological impacts of phycotoxins on grazers and the toxins distribution through marine food webs.

The brine shrimp *Artemia salina* is widely used for toxicity tests due to its widespread distribution, short life cycle, non-selective grazing, and sensitivity to toxic substances [38]. Therefore, *A*. *salina* seems to be a suitable model species to assess the toxicity of marine benthic dinoflagellates. The purpose of the present study was to evaluate the effects of the benthic dinoflagellates *P*. *lima*, *G*. *excentricus*, and *O*. cf. *ovata* on clearance and grazing rates, behavioral changes, and survival of *A*. *salina* during acute exposure. Acute toxicity assays were performed with cells in different growth phases of a batch culture (exponential and stationary) to assess differences in cell toxicity to brine shrimps.

## Material and methods

### Algae cultures

Clonal cultures of *Prorocentrum lim*a (strain UNR-01), *Ostreopsis* cf. *ovata* (strain UNR-05) and *Gambierdiscus excentricus* (strain UNR-08) used in this study were isolated from Armação dos Búzios (22°45ʹ18ʹʹ S, 41°54ʹ07ʹʹ W), Rio de Janeiro state as described in [5, 11, 39]. A non-toxic strain of the chlorophyte *Tetraselmis* sp. was isolated from Guanabara Bay, Rio de Janeiro state (22°15’-23°05’ S, 43°30’-42°30’ W). Scientific research and collecting permit authorizing field studies were obtained from Instituto Chico Mendes de Conservação da Biodiversidade (ICMBio), Brazilian Ministry of the Environment (permit number: 35192–3). No protected or endangered species was caught through field studies or used in experimental trials.

Benthic dinoflagellates were maintained in filtered seawater (glass-fiber filter, Millipore AP-40, Millipore Brazil) supplemented with L2 enrichment medium [40], modified by omitting silicate, nickel, vanadium and chromium; except for *O*. cf. *ovata* which was grown in L2/2 medium. Salinity was adjusted to 34 for all the cultures, except for *G*. *excentricus* that was cultivated at salinity 32. All stock cultures were kept in a temperature-controlled cabinet at 24 ± 2°C, with a 12:12 h dark-light cycle and photon flux density of 60 μmol m^-2^s^-1^ provided by cool-white fluorescent tubes. Photosynthetically active radiation was measured with a QSL-100 quantum sensor (Biospherical Instruments, San Diego, CA, USA).

The strain of *Prorocentrum lima* (UNR-01) used in the current study synthesizes mostly OA and small amounts of DTX-1 [13]. The *Gambierdiscus excentricus* strain (UNR-08) produces at least one MTX analog, while the evaluation of CTX production by this strain has not yet been completed (P Hess personal communication). The strain of *Ostreopsis* cf. *ovata* (UNR-05) has not been analyzed for toxin production. However, other strains of *O*. cf. *ovata* isolated from the same sampling location produce PLTX analogues (ovatoxins) [5].

Lugol preserved cells from the cultured strains were observed using light microscopy (Primovert, Zeiss, Germany) to measure the cells for the determination of cellular biovolume. Images were collected using an Axiocam Icc1digital camera (Zeiss, Germany), and cells (n = 40) were measured using the Axiovision software (Zeiss, Germany). Cellular biovolume was estimated using geometric shapes and mathematical equations suggested for each genus [41]: prolate spheroid for the chlorophyte and ellipsoid for the dinoflagellates. Cellular carbon content was calculated according to equations for dinoflagellates and chlorophytes [42].

### Experimental design

#### Feeding assays

Adult individuals of *A*. *salina* (7 mm ± 1.14) were acclimatized in experimental conditions in a temperature-controlled cabinet at 24 ± 2°C for 24 to 48 h and fed *ad libitum* with *Tetraselmis* sp. Feeding trials were performed in 6-well plates containing, each well, three individuals in 10 ml of filtered seawater (glass-fiber filter, Millipore AP-40, Millipore Brazil) at salinity 35.

Previous tests of *A*. *salina* grazing were carried out at various abundances of the non-toxic prey *Tetraselmis* sp. (150–720 cells ml^-1^) during an incubation interval that ranged from 30 min to 6 h. The results indicated the optimum incubation time (1 h) and *Tetraselmis* sp. abundance (~ 481 cells ml^-1^; 40 ng C ml^-1^) for feeding experiments in which the brine shrimps depleted 15–30% of cell abundances. Cell size of microalgae used in this study ranged from 13 μm (*Tetraselmis* sp.) to 78 μm (*G*. *excentricus*). Thus, the same carbon concentration was established in all treatments allowing the comparison of clearance and grazing rates of *A*. *salina* among different prey species. Cell abundances of microalgae that corresponded to the carbon concentration of 40 ng C ml^-1^, per well, were: *Tetraselmis* sp. (0.09 ng C cell^-1^, ~ 481 cells ml^-1^), *Prorocentrum lima* (2.15 ng C cell^-1^, ~ 19 cells ml^-1^), *Ostreopsis* cf. *ovata* (3.21 ng C cell^-1^, ~ 13 cells ml^-1^), and *Gambierdiscus excentricus* (10.95 ng C cell^-1^, ~ 4 cells ml^-1^). Microalgae species were harvested during exponential phase for feeding trials.

Each treatment containing the brine shrimps and one algal species was performed in triplicate, and the control containing solely algal cells without the brine shrimps was performed in duplicate. Aliquots of each replicate (1 ml) were collected using an automatic pipette at the beginning and after 1-h incubation. Cells were preserved with neutral Lugol's iodine solution for later cell counts using a Sedgewick-rafter chamber and observation in an inverted microscope (Primovert, Zeiss, Germany).

*Artemia salina* clearance (ml ind^-1^h^-1^; CR) and ingestion (ng C ind^-1^h^-1^; IR) rates were calculated by the ratio of natural log (ln) of initial and final carbon concentrations after 1-h incubation, and corrected by the carbon concentration in controls [43]. Clearance and ingestion rates were calculated for each replicate and, posteriorly, mean rates were calculated by treatment.

#### Acute toxicity assays

Adult individuals of *A*. *salina* (7 ± 1.14 mm) were acclimatized in the same experimental conditions described above for feeding experiments. Intoxication assays were performed in 6-well plates containing, each well, 10 ml of filtered seawater (glass-fiber filter, Millipore AP-40, Millipore Brazil) at salinity 35. Three individuals of *Artemia salina* were incubated for 7-h with each dinoflagellate species (*G*. *excentricus* or *O*. cf. *ovata* or *P*. *lima*) in abundances of 200 cells ml^-1^ [44]. Acute exposure of *A*. *salina* individuals to each toxic species was performed twice on independent samples, first with cells at exponential phase and in the second time with cells at stationary phase.

Dinoflagellate growth curves were previously determined [5, 11], and the non-harmful species *Tetraselmis* sp. (control) was kept and harvested in exponential growth for all trials. Toxic treatments were performed in triplicates and the non-toxic control in duplicate. In total, toxic treatments consisted of six different incubations: 1) *P*. *lima* in exponential phase, 2) *P*. *lima* in stationary phase, 3) *O*. cf. *ovata* in exponential phase, 4) *O*. cf. *ovata* in stationary phase, 5) *G*. *excentricus* in exponential phase, and 6) *G*. *excentricus* in stationary phase. Changes on swimming activity (actively swimming or motionless), position (individuals on the water column or at the bottom) and survival of individuals were verified after the first 30 min and after every hour of incubation.

Additional experiments were carried out to evaluate the effects of cell-free medium on brine shrimps survival. Cell-free medium were obtained by the filtration (glass-fiber filter, Millipore AP-40, Millipore Brazil) of *G*. *excentricus*, *O*. cf. *ovata* and *P*. *lima* cultures in abundances of 200 cells ml^-1^. Filtrates were used immediately. Incubations were performed in 6-well plates containing, each well, 10 ml of cell-free medium with six individuals of *A*. *salina* during 7-h. Each treatment of cell-free medium was performed in six replicates and the control (only filtered seawater) was performed in triplicate.

### Statistical analyses

One-way ANOVA was applied to evaluate the influence of four different preys on the ingestion and clearance rates of brine shrimps—categorical factor (*P*. *lima*, *O*. cf. *ovata*, *G*. *excentricus*, and *Tetraselmis* sp.). Normality and homogeneity of variances were assessed using Kolmogorov-Smirnov and Levene Test, respectively, and log-transformation was applied when necessary. Analyses were performed using the software Statistica 8.0 (StatSoft).

A redundancy analysis (RDA) was applied to data of *A*. *salina* individuals from toxic and non-toxic treatments using survival, position and swimming as explanatory variables, and exposure time (7 h) as covariable. A Monte Carlo permutation was used to test the significance of ordination model. The multivariate analysis was performed in the software CANOCO [45].

Generalized linear models (GLM) were applied to survival, swimming and position data (as dependent variables) of *A*. *salina* individuals from toxic treatments (as categorical factor) with exposure time (7-h) as continuous predictor in a full factorial design. Tukey test was applied whenever significant differences were detected by GLMs. A Kruskal-Wallis test was applied to survival data of *A*. *salina* individuals exposed to cell-free medium of dinoflagellate cultures. Analyses were performed using the software Statistica 8.0 (StatSoft).

Survival functions were estimated from the continuous survival time for 7-h brine shrimps exposure to the toxic dinoflagellates. The time when 50% of the individuals died (tD_50_) in each treatment was also estimated. A Kaplan-Meier log rank test (Mantel-Cox) was applied to test significant differences in the survivorship curve of *A*. *salina* among toxic treatments using the software GraphPad Prism 5.0 (GraphPad Software, San Diego—California). The Kaplan-Meier test takes into account the censored survivorship data [46].

## Results

### Feeding assays

**Fig 1:**
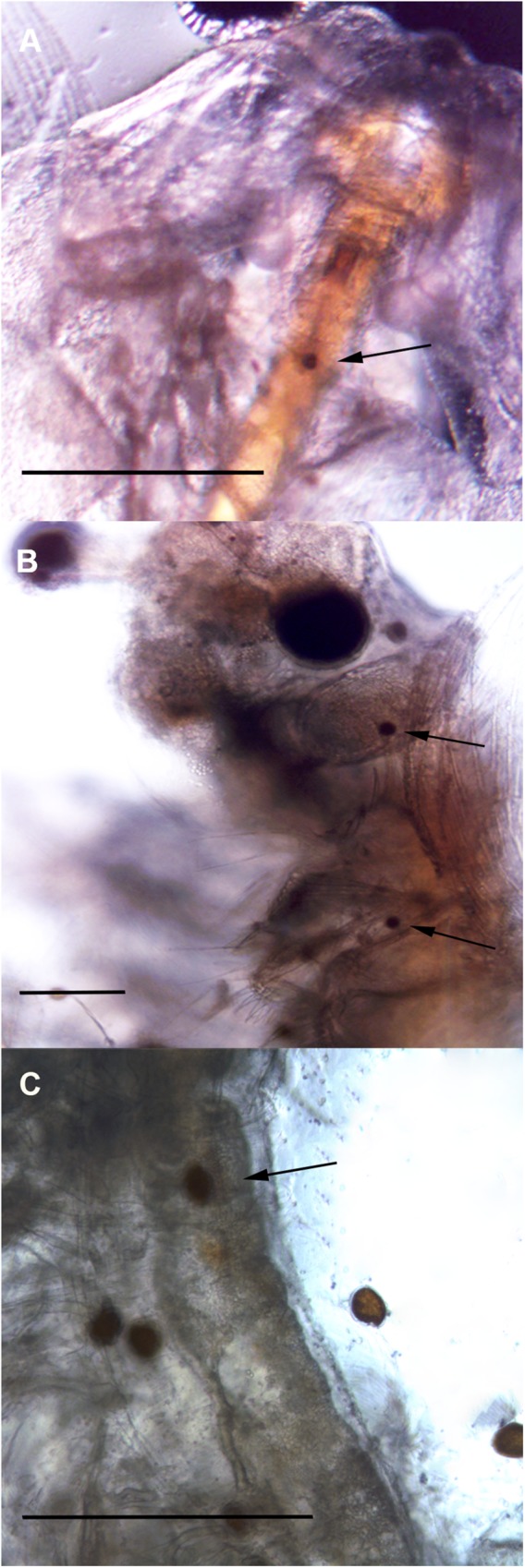
Toxic dinoflagellates in the digestive tract of *Artemia salina* individuals. Dinoflagellate cells are indicated by arrows (A) *Prorocentrum lima*, (B) *Ostreopsis* cf. *ovata*, and (C) *Gambierdiscus excentricus*. Scale bars: 200 μm.

The brine shrimps *Artemia salina* actively fed on all different preys offered to them (Fig 1), including the toxic species.

There was no significant difference in clearance (One-way ANOVA, F = 1.27, df = 3, p = 0.32) and ingestion rates (One-way ANOVA, F = 2.09, df = 3, p = 0.14) of the brine shrimps among the different treatments (Table 1). Adults of *A*. *salina* did not exhibit any unusual behavior when exposed to the toxic dinoflagellates during feeding assay.

**Table 1:** *Artemia salina* clearance and ingestion rates.

Preys	Clearance rates	Ingestion rates
	(ml ind^-1^ h^-1^)	(ng C ind^-1^ h^-1^)
***Tetraselmis* sp.**	1.12	50.55
**SD**	0.47	24.66
***Prorocentrum lima***	2.21	103.76
**SD**	1.25	64.88
***Ostreopsis* cf. *ovata***	1.19	127.64
**SD**	0.83	78.95
***Gambierdiscus excentricus***	1.60	54.34
**SD**	1.07	21.95

Mean and standard deviation (SD) values of clearance and ingestion rates of the brine shrimps on four prey species: the non-toxic *Tetraselmis* sp., and the toxic *Prorocentrum lima*, *Ostreopsis* cf. *ovata*, and *Gambierdiscus excentricus*.

### Acute toxicity assays

**Fig 2:**
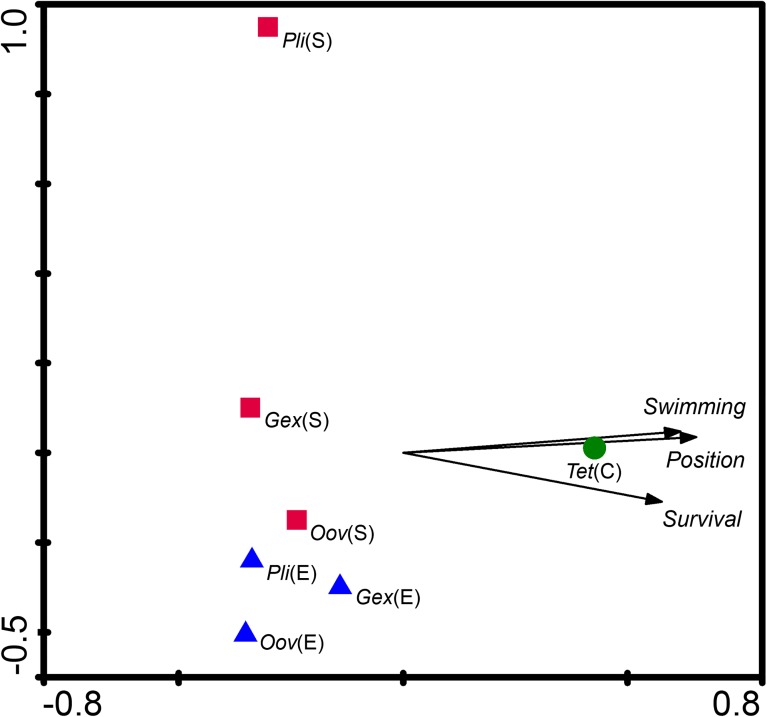
Survival and behavioral responses of *Artemia salina* exposed to toxic and non-toxic treatments. Biplot of survival and behavioral data of *A*. *salina* exposed to the non-toxic chlorophyte *Tetraselmis* sp. (*Tet*(C),●) and toxic dinoflagellates: *Prorocentrum lima* in exponential phase (*Pli*(E),▲), *P*. *lima* in stationary phase (*Pli*(S),■), *Ostreopsis* cf. *ovata* in exponential phase (*Oov*(E),▲), *O*. cf. *ovata* in stationary phase (*Oov*(S),■), *Gambierdiscus excentricus* in exponential phase (*Gex*(E), ▲), and *G*. *excentricus* in stationary phase (*Gex*(S), ■).

Redundancy analysis (RDA) applied to survival and behavioral data of *A*. *salina* clearly distinguished toxic treatments (negative coordinates on the first axis) from the non-toxic one (positive coordinates on the first axis; Fig 2). The canonical axes were statistically significant (p = 0.002); the first axis explained 98.5% total variance, while the second axis explained 1% total variance. The positive direction of the vectors swimming activity, water column position and survival confirms its positive correlation with samples from the non-toxic treatment (*Tetraselmis* sp.).

**Fig 3:**
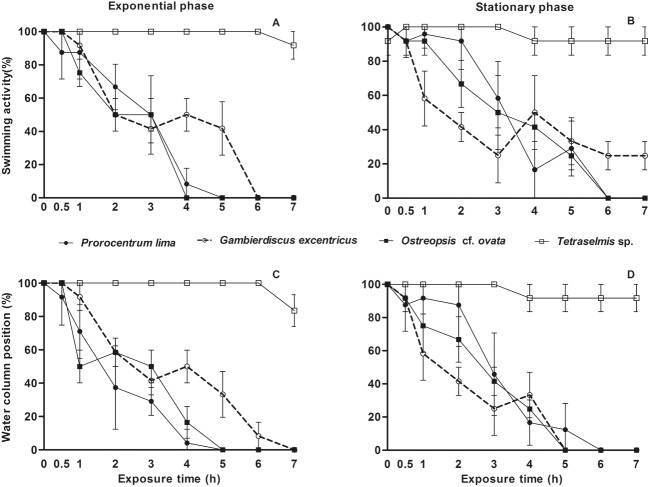
Behavioral changes in *Artemia salina* during acute exposure to toxic dinoflagellates. (A-B) Brine shrimps (%) actively swimming during exposure to dinoflagellate cells in exponential (A) and stationary (B) phase. (C-D) Brine shrimps (%) on the water column (in contrast to bottom position) during exposure to dinoflagellate cells in exponential (C) and stationary (D) phase. Different treatments are indicated by symbols: *P*. *lima* (•, full line), *G*. *excentricus* (○, dashed line), *O*. cf. *ovata* (▪, full line), and the chlorophyte *Tetraselmis* sp. (□, full line). The non-harmful chlorophyte used as control was kept and harvested in exponential phase for all the assays. Vertical bars: standard error considering four replicates.

The brine shrimp *A*. *salina* exposed to the toxic dinoflagellates *P*. *lima*, *O*. cf. *ovata* and *G*. *excentricus* showed abnormal behavior related to swimming activity and its position on the water column, independently of dinoflagellate growth phase (Fig 3). *Artemia salina* behavior was significantly affected by the time of exposure to toxic dinoflagellates (GLM, F_swimming =_ 370.6, F_position_ = 451.2, p<0.001), leading to the immobility of individuals at the bottom. Behavioral changes in the swimming activity and the position of the brine shrimps were noticed in the first 30-min incubations with dinoflagellates in stationary phase. After 1-h exposure to toxic cells, either in exponential or stationary phase, individuals have shown changes in swimming activity and in their position on the water column (Fig 3).

The swimming activity of *A*. *salina* was significantly affected by dinoflagellate species (GLM, F = 3.09, p = 0.01) and by the interaction between dinoflagellate species and time of exposure (GLM, F _=_ 4.9, p< 0.001). The *A*. *salina* activity was overall more affected by the exposure to *P*. *lima* and *O*. cf. *ovata* than to *G*. *excentricus* (Fig 3). No significant effect of dinoflagellate species (GLM, F = 1.36, p = 0.24) nor interaction between dinoflagellate and exposure time (GLM, F _=_ 0.64, p = 0.67) was detected on *A*. *salina* position on the water column.

**Fig 4:**
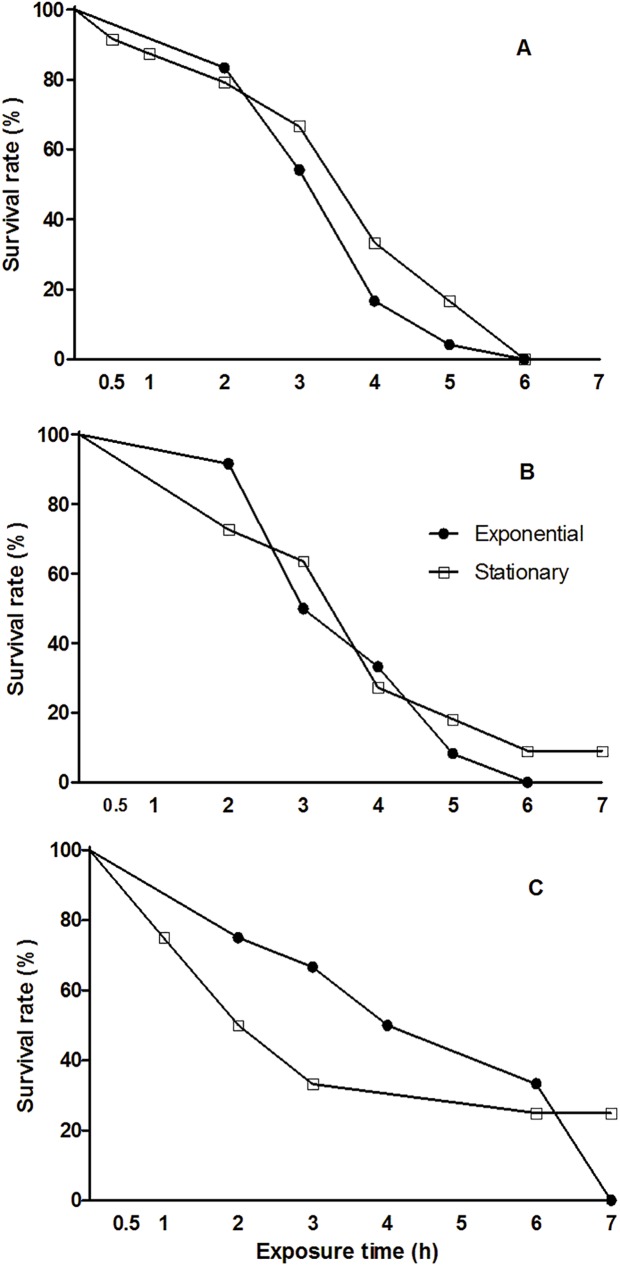
Survival rate curves of *Artemia salina* during acute exposure to the toxic benthic dinoflagellates in two growth phases: exponential (•) and stationary (□). (A) *Prorocentrum lima*, (B) *Ostreopsis* cf. *ovata*, and (C) *Gambierdiscus excentricus*.

The survival of *A*. *salina* was significantly affected by dinoflagellate species (GLM, F = 3.75, p = 0.003), by the exposure time (GLM, F = 430.74, p< 0.001), and by the interaction between dinoflagellate and exposure time (GLM, F = 4.41, p<0.001). The incubation of the brine shrimps with cell-free medium did not affect *A*. *salina* survivorship. There was no significant difference among treatments and control (Kruskal-Wallis, p = 0.23), survival rates ranged from ~92–100%: control (100%, ±0), and cell-free medium of *G*. *excentricus* (97.2%, ±6.8), *O*. cf. *ovata* (94.4%, ±8.6), and *P*. *lima* (91.7%, ±9.1). In contrast, the exposure to all the toxic dinoflagellate cells in exponential phase severely affected *A*. *salina* survival, with 100% mortality during assays (Fig 4). Survival rates of *A*. *salina* during exposure to *G*. *excentricus* and *O*. cf. *ovata* in stationary phase were 25% and 9%, respectively, after 7-h incubations. Only the exposure to *P*. *lima*, in both growth phases, lead to the death of all individuals within 6-h incubations (100% mortality, Fig 4).

Significant differences in the survival rates of *A*. *salina* were found for two pairwise comparisons using different species of dinoflagellate: (1) *P*. *lima* in stationary phase x *G*. *excentricus* in exponential phase (Tukey post-hoc test, p< 0.001), (2) *P*. *lima* in stationary phase x *O*. cf. *ovata* in stationary phase (Tukey post-hoc test, p = 0.016). During exposure to *P*. *lima* in stationary phase, less than 25% of *A*. *salina* individuals died in the first 2-h and, after a sharp decline in survival, all individuals were dead within 6-h incubation. In contrast, survival rate of *A*. *salina* exposed to *G*. *excentricus* cells in exponential phase slightly declined with over 30% survival after 6-h exposure. During exposure to *O*. cf. *ovata* cells in stationary phase, there was a sharp decline in *A*. *salina* survival in the first hours of incubation followed by a trend to stabilization close to 10% survival in the last hour (Fig 4). Significant differences related to the effect of growth phases on *A*. *salina* survival have been found only for *Gambierdiscus excentricus* (Tukey post-hoc test, p = 0.028). The survival of brine shrimps exposed to *G*. *excentricus* cells in exponential phase showed a steady decline until the end of incubation, reaching 100% mortality. In contrast, the exposure to *G*. *excentricus* in stationary phase led to a sharp reduction in survival within the first 3-h, with a loss of 25% of individuals per hour, followed by a stabilization in survival rate until the end of incubation with ~25% survival (Fig 4).

Two-hour incubation was the shortest time in which 50% of *A*. *salina* individuals have died (lower tD_50_) during exposure to *Gambierdiscus excentricus* in stationary phase (Table 2). Despite the quick effect of *G*. *excentricus* cells in stationary phase on brine shrimp survival, the highest survival rate (25%) of *A*. *salina* was found in the same treatment after 7-h exposure (Fig 4). The highest tD_50_ value was noticed for brine shrimps exposed to *G*. *excentricus* in exponential phase (4 h, Table 2). Intermediary and similar tD_50_ values were found in *P*. *lima* and *O*. cf. *ovata* treatments (Table 2).

**Table 2:** Time (h) when 50% of the *Artemia salina* died (tD_50_) during exposure to toxic dinoflagellates in two cell growth phases.

Growth phase	*Prorocentrum lima*	*Ostreopsis* cf. *ovata*	*Gambierdiscus excentricus*
Exponential	3.1	3.0	4.0
Stationary	3.5	3.4	2.0

## Discussion

The four microalgae offered as prey have different morphological and toxicological characteristics that would account for differences in clearance and ingestion rates of potential grazers. However, the brine shrimp *Artemia salina* actively fed on the different preys offered to them: three species of toxic benthic dinoflagellates and one non-harmful chlorophyte. No significant differences in clearance and ingestion rates were noticed among the four treatments. Thus, clearance and ingestion rates of brine shrimps seem to be unaffected by differences in cell size in the range 13–78 μm. In a previous study, clearance rates of *A*. *salina* on the dinoflagellate *Cochlodinium polykrikoides* were three-to-four times faster than clearance rates on the cryptophyte *Rhodomonas salina* suggesting preferences for prey in the range 2–35 μm and for dinoflagellate as prey [47].

In addition, neither preference nor avoidance of toxic prey has been shown by the brine shrimps. The clearance and ingestion rates of brine shrimps were not affected by the toxicity of the dinoflagellates during the short-term experiment of 1-h incubation. This is probably due to the low cell abundances offered to them ~4–19 cells ml^-1^. Similarly, no preference has been shown by the copepods *Acartia tonsa* and *Temora longicornis* with similar ingestion rates on diatoms, the toxic *Pseudo-nitzchia multiseries* and the non-toxic *P*. *pungens* [48]. In the same way, *A*. *salina* grazed on the paralytic shellfish toxins (PSTs) producer *Alexandrium fundyense* at rates similar to that displayed for the non-harmful cryptophyte *Rhodomonas salina* [47]. However, brine shrimp feeding rates on *A*. *fundyense* decreased when dinoflagellate abundances increased, while an opposite trend was observed in feeding rates on the non-harmful species [47]. Lower feeding rates of *A*. *salina* were observed when grazing on toxic *Alexandrium* species (*A*. *catenella*, *A*. *minutum* and *A*. *tamarense*) compared to grazing on a mixture of toxic and non-toxic dinoflagellates (*A*. *tamarense* + *Prorocentrum donghaiense*), and solely on the non-toxic *P*. *donghaiense* [49].

The threat of natural marine toxins increases considerable when bioaccumulation is considered along the food chains [50]. As the brine shrimps grazed on the harmful dinoflagellates, ingested cells that remained in *A*. *salina* tract can be transferred to secondary consumers as demonstrated for *Gambierdiscus* cells [31]. The toxic compounds may also be incorporated and accumulated into individuals, as previously described for *A*. *salina* exposed to PSTs [51] and microcystin producers [52]. Phycotoxins retained into individuals may be transformed by detoxification processes, such as the glutathione S-transferase described in *A*. *salina*, responsible for detoxification of various toxic compounds [53]. Crustaceans are key prey for marine species of higher trophic levels and perform daily and seasonal migration; therefore crustaceans that graze on toxic dinoflagellates (as *Artemia salina* shown in the present study) can act as vectors of dinoflagellate toxins in marine food webs [54].

The current intoxication trials contribute to increase the knowledge about direct toxic effects of benthic dinoflagellates on a marine invertebrate widely used as model organism in bioassays—*Artemia salina* [55]. The toxic *Prorocentrum lima*, *Ostreopsis* cf. *ovata* and *Gambierdiscus excentricus* isolated from tropical marine systems caused harmful effects on behavior and affected the survival of *A*. *salina* at abundances of 200 cells ml^-1^. The harmful effects on behavior and survival of the brine shrimps were demonstrated by multivariate analysis (RDA) in which toxic treatments were clearly distinguished from the non-toxic control that was positively correlated with swimming activity, water column position and survival of *A*. *salina*.

Immobility and mortality were used as endpoints to measure the toxicity of the three benthic dinoflagellates to adults of *A*. *salina*. Healthy *Artemia* individuals are active swimmers, thus, alterations in swimming activity are valid as behavioral endpoints to detect stress at sub-lethal concentrations of toxic compounds [56]. The brine shrimps exposed to the toxic dinoflagellates exhibited reduced swimming activity, ultimately immobility. Swimming behavior has a direct impact on zooplankton dispersal, encounter with prey and predators, and vulnerability to predation which determine the propensity of individuals to graze on harmful species and transfer phycotoxins through the food web [36]. Only ~20% of *A*. *salina* individuals exposed to *G*. *excentricus* cells in stationary phase showed any movement after 7-h incubation.

Besides, brine shrimp exposure to *O*. cf. *ovata* and *P*. *lima* induced the loss of movement or death of all the individuals until the end of incubation. Similarly, adult females of the copepod *Tigriopus japonicus* exposed to *Fukuyoa* sp. (as *Gambierdiscus* cf. *yasumotoi*) showed a decrease in activity, loss of motor control, and abnormal swimming [57]. However, female copepods reached 100% immobility only after 12-d exposure to 200 cells ml^-1^ of *Fukuyoa* sp. (as *Gambierdiscus* cf. *yasumotoi*) [57], the same abundance used in acute toxicity assays of the present study. Abnormal behavior exhibited by the crustaceans exposed to toxic dinoflagellates increases their vulnerability to predation [58–59]. In addition, a reduction in locomotion activity may affect the vertical migration of nauplii [60].

The present results showed that the toxic dinoflagellates *P*. *lima* and *O*. cf. *ovata* were able to induce similar effects on behavior, particularly swimming activity, and survival of the brine shrimp *A*. *salina*. The exposure to these two dinoflagellates caused a sharp reduction on swimming activity followed by an increase in mortality of *A*. *salina* adults. The OA and DTX-1 toxins produced by *P*. *lima* probably lead to physiological disturbances in brine shrimps related to loss of body fluids and lack of physiological control of fluid dynamics, as previously observed in affected organisms [44, 61]. Similarly, PLTX analogues potentially produced by *O*. cf. *ovata* alter the mechanisms of ion homeostasis [62], with disruption of cell membrane functions and loss of ion regulation [38]. In the present study, the adverse effects on *A*. *salina* induced by the exposure to *P*. *lima* and *O*. cf. *ovata* seem to be thus related to brine shrimp ion regulation.

The toxic benthic dinoflagellates significantly affected the survival rates of *A*. *salina* during acute exposure. All the brine shrimps exposed to the three toxic dinoflagellates in exponential phase died, while the mortality of brine shrimps exposed to cells in stationary phase varied according to dinoflagellate species: 100% (*P*. *lima*), 91% (*O*. cf. *ovata*), and 75% (*G*. *excentricus*). However, the cell-free medium did not significantly affect *A*. *salina* survival during acute exposure. Cell-free medium from cultures with higher abundances of *O*. cf. *ovata* (~4000 cells ml^-1^) [38] were harmful to nauplii of *A*. *salina*. In the present study, the high survival of the brine shrimps exposed to cell-free medium of *G*. *excentricus*, *O*. cf. *ovata* and *P*. *lima* confirms that the concentration of diluted cell exudates was within *A*. *salina* tolerance and did not compromise their survival.

*Artemia salina* exhibited similar sensitivity to *Prorocentrum lima* and *Ostreopsis* cf. *ovata* acute exposure; the same pattern seen for behavioral responses. Adults of *A*. *salina* showed high sensitivity to *P*. *lima* either in exponential or stationary growth phases (respectively, tD_50_ = 3.1 and 3.5h) and to *O*. cf. *ovata*, particularly in exponential growth phase (tD_50_ = 3h), with 100% mortality after 6-h exposure. Conversely, minimal mortalities of *A*. *salina* adults have been reported after exposure to *P*. *lima* and *P*. *concavum* in abundances up to 1000 cells individual^-1^[31]. Previous studies on *A*. *salina* larvae have demonstrated high sensitivity of nauplii to both *P*. *lima* (tD_50_ = 1.7h) [44] and *O*. cf. *ovata* cells [38, 63]. In addition, *A*. *salina* nauplii were reported to be extremely sensitive to *O*. cf. *ovata* cells with half maximum effective concentration (EC_50_) ranging from 6 to 24 cells ml^-1^ [63]. Exposure of different crustaceans to *O*. cf. *ovata* cells revealed that *A*. *salina* larvae have the highest sensitivity among them, with a half lethal concentration (LC_50_) lower than 4 cells ml^-1^ [38]. *Artemia salina* has also showed high sensitivity to other *Ostreopsis* species, the PTX-like producer *O*. *siamensis* [64]. The sensitivity exhibited by *A*. *salina* in the two stages of its life cycle (nauplius and adult) to these toxic dinoflagellates confirms this crustacean as a suitable model species to assess the toxicity of marine harmful algae.

The dinoflagellate *Gambierdiscus excentricus* rapidly affected the swimming activity, water column position and survival of exposed-individuals of *A*. *salina* in the present assays. A short stabilization of effects was observed after 3-h exposure, followed by a reduction in the percentage of individuals swimming on the water column as well as in the survival of the brine shrimps between 5 to 7-h exposure. Maitotoxins (MTX) alter ion transport systems causing an increase in free intracellular Ca^2+^ [65] while ciguatoxins (CTXs) are neurotoxins that bind to the voltage-sensitive sodium channels on cell membranes [66–67]. The toxic effects of ciguatoxins seem to be similar to saxitoxins effects, the neurotoxins produced by the planktonic dinoflagellate *Alexandrium* spp. that act on the voltage-gated Na^+^ channel of nerve cells, leading to loss of motor control and abnormal swimming on copepod [68]. Exposure to *Gambierdiscus* sp. (as *Gambierdiscus toxicus*) and *Fukuyoa* sp. (as *Gambierdiscus* cf. *yasumotoi*) was lethal to the adult brine shrimp *Artemia* spp. [31] and the copepod *Tigriopus japonicus* [57]. In the present study, adults of *A*. *salina* showed high sensitivity to *G*. *excentricus* in both growth phases (respectively, tD_50_ = 2h and 4h) at 200 cells ml^-1^, with 100% mortality after 7-h exposure to cells in exponential phase. Similarly, strains of *Gambierdiscus* sp. (as *G*. *toxicus*) showed high toxicity to *Artemia* with median lethal dose (LD_50_) from 2.8 to 104.5 cells individual^-1^ [31]. Lower effects on survival, with less than 60% of individuals dying in 6-d exposure were found in copepods exposed to *Fukuyoa* sp. (as *Gambierdiscus* cf. *yasumotoi*) at 200 cells ml^-1^ [57], the same abundance used in the present study. However, a suppression in gene expression related to stress or detoxification has suggested a deficiency in detoxification of ciguatoxins by these copepods [57]. Differential responses in zooplankton sensitivity may be related to the toxicity of the species *Fukuyoa* sp. [57] and *G*. *excentricus* (current study), as well as the feeding selectivity against harmful prey or sensitivity of the tested species (*T*. *japonicus* and *A*. *salina*, respectively).

The toxin content or cell quota may vary along the growth phases of a dinoflagellate species in a batch culture and may be lower during the exponential phase relative to the stationary and the declining phase, as observed for *O*. cf. *ovata* [69–70]. In eight species of *Gambierdiscus*, MTX-related hemolytic activity increased from log phase (exponential) to late log-early stationary phase, but then declined in mid-stationary phase, although no significant differences in toxicity were observed among growth phases [66]. Further study is needed to determine if the amount of toxin per cell and toxin profile varies along the growth phases of *Gambierdiscus* species. In the current study, significant differences between the toxic effects of dinoflagellate growth phases was solely found in survival rates of *A*. *salina* exposed to *G*. *excentricus*. Exposure to cells in stationary phase induced an initial quick response on *A*. *salina* survival, with high mortality of 50% within the first 3-h incubation, and 75% mortality at the end of 7-h incubation. In contrast, *G*. *excentricus* cells in exponential phase induced a steady decrease in survival rates and caused 100% mortality of brine shrimps at the end of incubation. The difference in *A*. *salina* survival rates may be related to an effect of the production of different toxin congeners with varying toxicities for the brine shrimps in each growth phase. Moreover, *G*. *excentricus* is a large thecate (with cellulose plates) dinoflagellate and it is likely that aged cells (in stationary phase) are thicker than actively growing (in exponential phase) cells. As cells enter the stationary phase, the growth rate diminishes, but cells may continue to expand in size. In many armored species, the edges of each thecal plate overlap, sliding apart as the cells increase in size, likely producing a more robust theca. The thicker and more robust aged cell is possibly more resistant to the digestion by *A*. *salina*, which will affect toxin assimilation rate and ultimately the survival of potential grazers.

Finally, acute exposure to the toxic benthic dinoflagellates *Prorocentrum lima*, *Ostreopsis* cf. *ovata* and *Gambierdiscus excentricus* isolated from tropical marine systems induced severe effects on behavior and survival of adult individuals of *Artemia salina*. Further studies are necessary to identify the different modes of action of diverse toxic compounds produced by benthic dinoflagellates on their primary grazers. Negative effects related to the toxicity of benthic dinoflagellates are expected on diverse grazers making them more vulnerable to predation and vectors of toxins through the marine food webs.

## Supporting information

S1 File (XLS)
